# 

**Published:** 1888-05

**Authors:** 


CONTENTS

VOL. 35 NO. 5.

| | PAGE. |
|---|---|
| The Labor Question | 97 |
| History of Mind Cure | 99 |
| A Lofty Imagination | 101 |
| Cruelty in the Crib | 102 |
| Fashion and Common Sense | 102 |
| The banana Tree | 105 |
| Boiling as a means of Keeping Milk | 105 |
| Syrian Wives | 106 |
| The Uses of Forrests | 107 |
| Alcohol | 108 |
| Hypatia | 110 |
| A Singular Pet | 111 |
| Animal Intelligence | 112 |
| Mysterious Light | 113 |
| Spiders Once More | 114 |
| Rattle Snake Oil | 115 |
| Book Notices | 116 |
| Household | 117 |
| Miscellaneous | 119 |

INDEX TO ADVERTISEMENTS OF HALF A PAGE AND OVER.

| | PAGE. |
|---|---|
| Crystal Pepsin Tablets | I |
| Stevensville Mills | II |
| The Tea Cure | II |
| The American Oxygen Association | III |
| Hollow Suppositories | IV |
| Lactopeptine | V |
| New York to the Front | VI |
| Bovinine | VIII |
| Premiums | X |
| Celerine | XI |

